# Synergistic Effects of Graphene Oxide and Pesticides on Fall Armyworm, *Spodoptera frugiperda*

**DOI:** 10.3390/nano12223985

**Published:** 2022-11-12

**Authors:** Xue Li, Qinying Wang, Xiuping Wang, Zhenying Wang

**Affiliations:** 1 State Key Laboratory for Biology of Plant Diseases and Insect Pests, Institute of Plant Protection, Chinese Academy of Agricultural Sciences, Beijing 100193, China; 2 Plant Protection College, Hebei Agricultural University, Baoding 071000, China; 3 Analysis and Testing Center, Hebei Normal University of Science and Technology, Qinhuangdao 066000, China

**Keywords:** graphene oxide, pesticide, *Spodoptera frugiperda*, toxicity, synergistic effect

## Abstract

Fall armyworm *Spodoptera frugiperda*, a native insect pest in tropical and subtropical America, has rapidly spread to most parts of China and become a major pest of corn and other crops since invading in early January 2019. As an emergency and important control measure, chemical control of *S. frugiperda* has the advantages of quick effect and low cost. However, long-term and large-scale use of pesticides might pollute the environment and increase pest resistance. By improving the control effect and reducing the dosage of chemical pesticides, graphene oxide (GO) is used synergistically with insecticides to increase control efficacy to achieve low-cost and sustainable management of insect pests as a new type of synergist. In this study, graphene oxide was compounded with insecticides to form nanocomposites. To clarify pest physiological responses, the laboratory toxicity of graphene oxide-insecticide nanocomposites was measured on the larvae of *S. frugiperda*. The results demonstrated that GO could enhance the activity of four selected pesticides: chlorantraniliprole (Chl), beta cypermethrin (Bet), methoxyhydrazide (Met) and spinetoram (Spi). Compared with pesticides alone, the toxicity of Chl-GO, Bet-GO, Met-GO and Spi-GO mixtures to the third instar larvae of *S. frugiperda* increased by 1.56, 1.54, 2.53 and 1.74 times, respectively. The easy preparation and higher bioactivity of GO-pesticide nanocomposites indicated their promising application potential in pest control.

## 1. Introduction

Fall armyworm, *Spodoptera frugiperda* (J.E. Smith, 1797) (Lepidoptera: Noctuidae), originated in tropical and subtropical America [1,2] and is a major pest on global alert issued by the Food and Agriculture Organization of the United Nations (FAO) [3]. Since 2016, *S. frugiperda* has invaded more than 60 countries and regions in Africa, Asia and Oceania [4]. So far, *S. frugiperda* is globally distributed in more than 120 countries and regions [5]. As a polyphagous insect pest, it can attack more than 350 species of host plants, including a large number of cultivated plant species [6], such as *Saccharum officinarum* [7], *Zea mays*, *Oryza sativa*, *Arachis hypogaea*, *Sorghum bicolor* (L.) *Moench*, *Triticum aestivum*, *Gossypium* spp., *Beta vulgaris* and *Nicotiana tabacum* [6,8,9,10]. The damage of *S. frugiperda* on crop plants can result in the decline of plant yield and may lead to no harvest. Chemical control is now the main emergency management of *S. frugiperda*. It plays an important role in restraining the pest population and reducing crop damage [11,12]. However, long-term and large-scale application of pesticides may lead to pest resistance. *S. frugiperda*, as the main food crop pest in recent years, poses a serious threat to China’s food security [13]. Therefore, efficient control of *S. frugiperda* has become one of the hot issues of plant protection at home and abroad in recent years.

Recently, a carbon nanomaterial-based pesticide delivery system has drawn great attention for high pesticide loading capacity, excellent stability and bioactivity, easy preparation and industrialization potential [14,15,16]. As one of the viable nano-carriers, graphene oxide (GO) is a two-dimensional carbon nanomaterial with honeycomb monolayer structure composed of sp^2^ carbon atoms. It possesses plenty of oxygen-containing polar functionalities, such as carbonyl, epoxide, carboxyl and hydroxyl groups [17,18,19,20]. Its oxygen functional groups increase with improved properties and easier surface modification [21,22,23,24]. It has a large surface area ratio and can be used to construct drug delivery systems. Besides, after combining graphene oxide with drug molecules through covalent or non-covalent bonding, it can expect a high drug loading rate, good water solubility and remarkably targeted delivery [23]. Recently, the application of GO in pesticides has attracted great research interests. It has been demonstrated that GO has no toxic effect on Asian corn borer (ACB), *Ostrinia furnacalis* (Guenée), but could significantly improve the larval survival rate, larval weight, duration rate of ACB and shorten the larval development period. It would not cause negative effects on the emergence rate and fecundity [23]. The nanocomposite formed by GO and acaricide is adsorbed on the body wall of *Tetranychus cinnabarinus* and *Tetranychus runcates* to improve the utilization efficiency of pesticides [16,25]. The combination of GO with water insoluble pesticides through covalent or non-covalent bonding can synthesize GO-pesticides with a higher pesticide loading rate and better targeted delivery [26,27]. These findings imply GO to be a promising carrier for pesticides in pesticide formulation. Spinetoram (Spi), methoxyhydrazide (Met), betacypermethrin (Bet) and chlorantraniliprole (Chl) are the recommended pesticides for emergency control of *S. frugiperda* by Ministry of Agriculture and Rural Affairs of China in 2019 and 2020 [28]. In this study, we prepared new GO-pesticide nanocomposites by combining GO with these recommended insecticides for better control of *S. frugiperda*. The aims of this study are: (1) to determine the optimal combination ratio of GO and pesticide; (2) to characterize the morphology, drug loading and thermal stability of GO-pesticide nanocomposites; (3) to detect the bioactivity of GO-pesticide nanocomposites against the 3rd instar larvae of *S. frugiperda*. The results will provide new ways to control *S. frugiperda* by the new formulation of nanopesticides.

## 2. Materials and Methods

### 2.1. Insect Rearing

*S. frugiperda* larvae were obtained from a laboratory colony from the Institute of Plant Protection, Chinese Academy of Agricultural Sciences. This colony was originally collected from a winter corn field in Mangshi, Dehong Prefecture, Yunnan Province (24.42° N, 98.60° E). *S. frugiperda* larvae were then reared for three to four generations on an insecticide-free artificial diet with 65 ± 5% RH, 26.5 ± 0.5 °C under a 14 h L:10 h D photoperiod. All the third instar larvae were starved for two hours prior to experiments.

### 2.2. Materials

GO was purchased from MACKLIN Biochemical Technology (Shanghai, China) (thickness 0.6 nm, length 0.8 μm, purity 98.5%). Technical-grade spinetoram (98.0%), chlorantraniliprole (99.0%) and methoxyhydrazide (98.0%) were supplied by Corteva Agriscience (Shanghai, China). Beta cypermethrin (97.0%) was supplied by Pesticide Pilot Plant of Chinese Academy of Agricultural Sciences (Langfang, China). Dimethyl sulfoxide (DMSO) was purchased from Beijing Exelon Biotechnology (Beijing, China). General Purpose Lepidoptera Diet (Product#F9772, Quantity:20 L, Lot:051721-01, Frontier Agricultural Sciences, https://www.insectrearing.com/?s=F9772, accessed on 25 October 2022).

### 2.3. Larval Toxicity Bioassay

The laboratory surface coating method [29] was adopted from Gao et al. to determine the insecticidal activity of Chl-GO, Spi-GO, Met-GO and Bet-GO against the third instar larvae of *S. frugiperda*. The pesticide (Chl, Spi, Met and Bet) alone and combined with GO were assayed against *S. frugiperda*, as described in Table 1. First, we added the non-coagulated artificial diet to a 24-well cell culture dish and set it naturally at room temperature. A series of gradient concentration reagents were added onto the surface with a pipette, and the volume of reagents added per well was 50 μL. After the surface dried, artificial diets containing different concentrations of insecticides were fed. All experiments were carried out in the growth chamber under the following conditions: 65 ± 5% RH, 26.5 ± 0.5 °C and 14 h L:10 h D. The distilled water containing 0.1% Tween-80 and 2%DMSO were set as controls. We then picked third instar larvae individually to each hole and covered breathable plastic film to prevent escape. Each concentration was set up three replicates, 24 insects per replicate and larvae survival was checked after 48 h. No reaction was considered as dead when touching insects with a brush.

### 2.4. Preparation of GO-Pesticide Nanocomposites

Different insecticides were dispersed in the mixture containing 2 mL DMSO and 0.1% Tween-80 aqueous solution. Then GO was added to make the mass ratio of GO to each pesticide by the ratios of 1:9, 2:8, 3:7, 4:6, 5:5, 6:4, 7:3, 8:2 and 9:1. The morphology of GO and GO-pesticide nanocomposites was examined by scanning electron microscopy (SEM, Regulus 8100). The solid sample was fixed on a conductive tape and sprayed with gold with a thickness of about 7 nm in a vacuum environment. The adsorption of graphene oxide and pesticides was visually observed at a voltage of 10 KV. Infrared absorption spectra of GO and GO-pesticide nanocomposites were measured on a Fourier transform infrared (FT-IR) spectroscope (Bruker, TENSOR-27 Karlsruhe, Germany)at room temperature. Thermogravimetric analysis (TGA) was performed with an STA 409 PC (Nietzsche, Germany) from room temperature to 700 °C at a heating rate of 10 °C min^−1^ under N_2_. X-ray diffraction (XRD) was performed on SmartLab 9 kW, which was equipped with Cu-Kα radiation of 1.5406 nm. The XRD patterns were recorded over 2θ region of 0 to 100° with a scanning speed set at 2° per min.

### 2.5. HPLC Analysis

The loading amount of pesticides on the surface of GO was measured by HPLC (Diane U3000 Thermofisher, USA), which was employed by using ZORBAX Eclipse XDB-C18 column with the size of 150 mm × 4.6 mm × 5 μm (Spi), 250 mm × 4.6 mm × 5 μm (Bet), 150 mm × 4.6 mm × 5 μm (Met), 100 mm × 2.1 mm × 3.5 μm (Chl); with UV detection at 254 nm (Spi), 278 nm (Bet), 245 nm (Met) or 270 nm (Chl). A flow rate of 1 mL min^−1^ was used with a mobile phase composition of acetonitrile and water 98:2 *v/v* (Spi), 77:23 *v/v* (Bet), methanol and water 65:35 *v/v* (Met), 95:5 *v/v* (Chl); and the injection volume was 10 μL (Spi, Bet and Chl) and 3 μL (Met). For determination of the loading amount, the prepared GO–pesticide nanocomposites were dissolved in deionized water. The pesticide loading content (LC) was obtained with the following equation [25]:

LC (%) = *W*_pesticide_/*W*_GO_ × 100 (1)

where *W*_pesticide_ is the weight of pesticide loaded on GO (mg), and *W*_GO_ is the weight of GO (mg).

### 2.6. Statistical Analysis

LC_50_ values for insecticide were calculated using the Polo Plus software (v 1.0, LeOra Software, Parma, MO, USA). TGA and FT-IR curves of each reagent were drawn using Origin Pro 9.0. One-way analysis of variance (ANOVA) was completed in SAS using the Fisher’s protected LSD test. GraphPad Prism 8.0.2 was used to draw the chart, and all values in the tables were expressed as the mean. The synergy ratio (SR) of GO to pesticides was calculated as follows:

SR = LC_50 pesticides_/LC_50 pesticides-GO_ (2)

0.5 < Values of SR < 1.5 indicate interactions.

Value of SR > 1.5 indicate synergistic interactions [25].

## 3. Results

### 3.1. SEM and EDS Analysis

**Figure 1:**
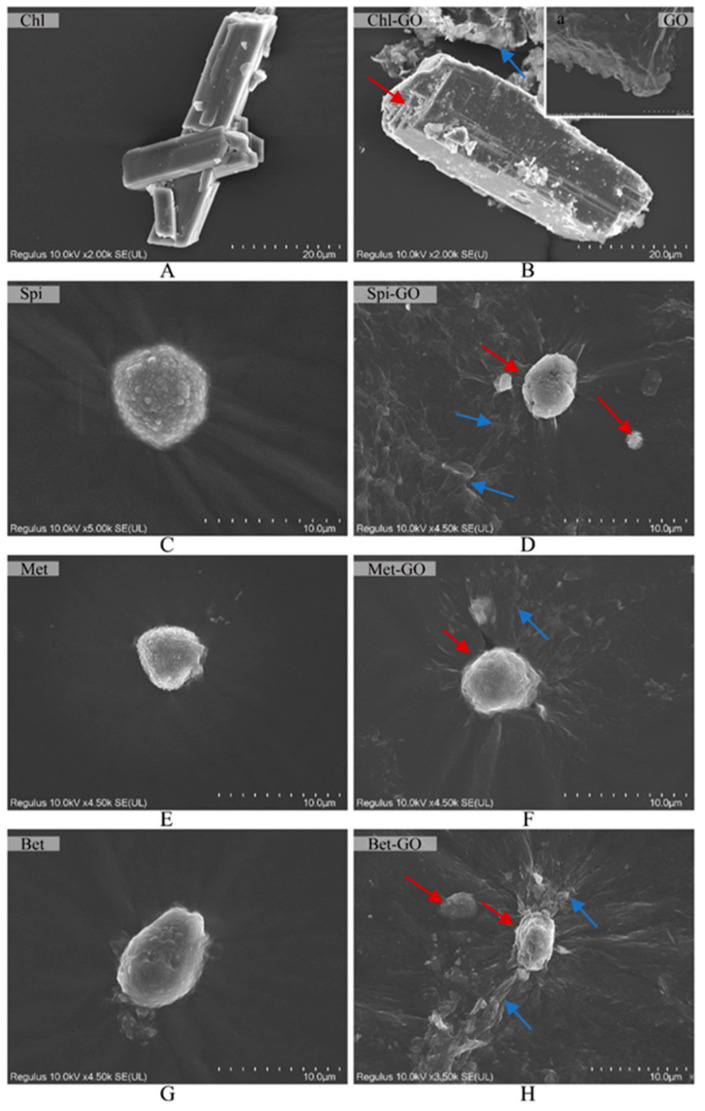
SEM images of GO, pesticide and GO-pesticide nanocomposites. GO (**Ba**), Chl (**A**), Spi (**C**), Met (**E**), Bet (**G**), Chl-GO at 5:5 (**B**), Spi-GO at 3:7 (**D**), Met-GO at 3:7 (**F**) and Bet-GO at 1:9 (**H**). Red arrows indicate insecticide, blue arrows indicate GO.

GO had an excellent adsorption capacity and good water dispersion performance as an ideal pesticide carrier [30]. To explore the structural characteristics of GO-pesticides, the surface structural characteristics of GO, pesticide and GO combined pesticide complex were analyzed using SEM. GO showed an aggregated multilayer sheet structure with a typical rough and folded appearance (Figure 1(Ba)) [31]. This observation showed that graphene was fully stripped during chemical oxidation. The surface of the four pesticides was smooth in different shapes. Among the shapes were the crystals with hexahedral structures of Chl (Figure 1A) and irregular spherical shapes with smooth surfaces in the other three (Figure 1C,E,G). After loading GO, numerous folded patellar crystals (Figure 1B,D,F,H) were observed on the surface, indicating that the crystals of Chl, Bet, Spi and Met were adsorbed on the surface by GO flakes.

**Figure 2:**
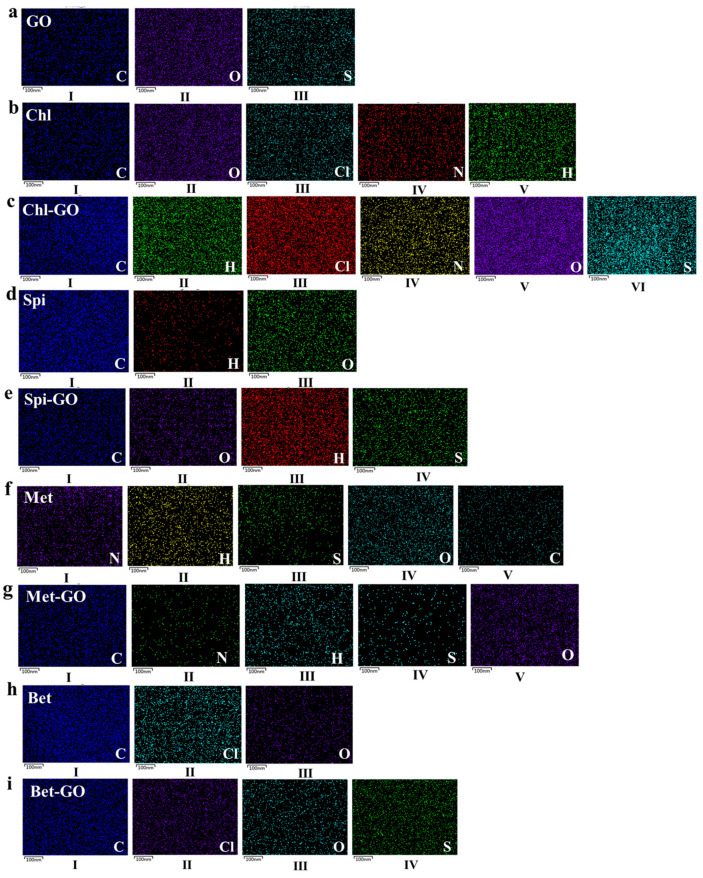
SEM-EDS of GO after GO (**a**), Chl (**b**), Spi (**d**), Met (**f**), Bet(**h**), Chl-GO (**c**), Spi-GO (**e**), Met-GO (**g**) and Bet-GO (**i**) adsorption.

The morphology of GO, pesticides, and pesticide-GO was analyzed by TEM. The results of the sample by EDS are shown in Figure 2 to further explore the chemical composition of each drug sample. It can be seen that the main chemical composition of GO elements are C, O, and S, among which the content of carbon was the most, indicating that GO contained a large amount of carbon. The content of each element increased after physical adsorption with pesticides, showing an additive effect.

The structural elements of GO were mainly C and O, with a small amount of S and no N. The constituent elements of Chl included C, H, N, O and Cl. As shown in the Figure 2b, the EDS image of GO after adsorption of Chl showed C, H, Cl, N, O and S. In Figure 2c, adding the characteristic elements of Chl suggested GO adsorbing. The other three pesticides had similar results. The constituent elements of Spi included C, O and H (Figure 2d). The constituent elements of Met included C, H, N, O and S (Figure 2f). Bet including C, Cl and O proved that GO adsorbs Spi, Met and Bet (Figure 2h).

### 3.2. XRD of GO-Pesticide Nanocomposites

**Figure 3:**
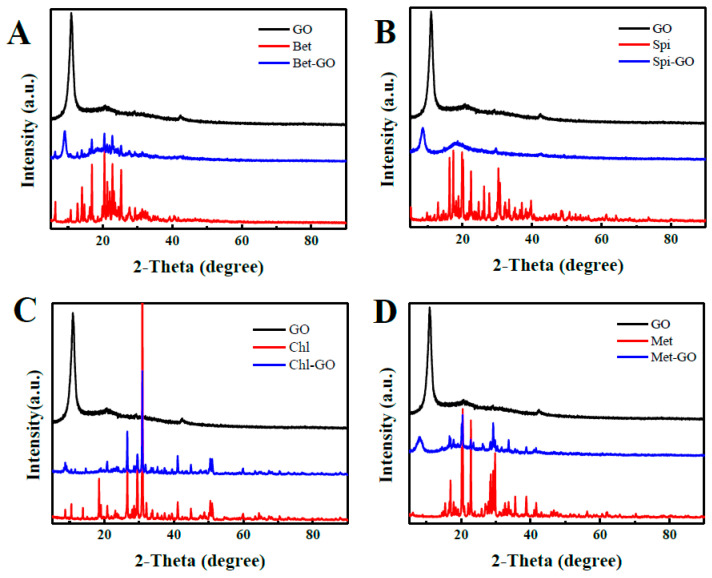
XRD curves of GO and pesticide-GO nanocomposites, Bet and Bet-GO (1:9) (**A**), Spi and Spi-GO (3:7) (**B**), Chl and Chl-GO (5:5) (**C**), Met and Met-GO (3:7) (**D**).

Figure 3 showed the XRD patterns of insecticides, GO and insecticide-GO. GO had a typical broad characteristic diffraction peak at 2θ = 10.7°, which corresponded to the characteristic diffraction peak of the (002) crystal plane of GO. It indicated that after graphite oxidized, hydroxyl (-OH), carboxyl (-COOH), ring Oxygen-containing functional groups such as oxygen (C-O-C) were introduced into the surface or edge of the graphite sheet, and the graphite was converted into GO. The characteristic peaks of pesticides can be clearly seen in the XRD of GO-insecticide, and the characteristic diffraction peaks of GO in Bet-GO, Spi-GO, Chl-GO and Met-GO are shifted to 9.01°, 8.6°, 8.72°and 8.17°, respectively. The peak intensity increases as well. The increase in peak intensity indicated that the addition of GO to the pesticide increases the crystallinity [27]. The results of the XRD analysis highlighted the marginal increase in the insecticide diffraction at 2θ after adding GO to the insecticide. This increment may affect the adsorption capacity of the nanocomposites formed by the combination of pesticides and GO to the pesticides.

### 3.3. Thermal Stability Analysis of GO-Pesticide Nanocomposites

**Figure 4:**
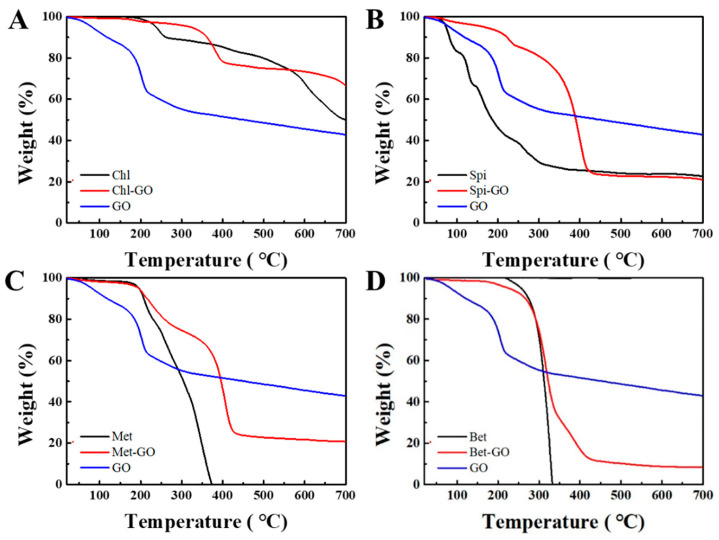
TGA curves of GO and pesticide-GO nanocomposites, Chl-GO (5:5) (**A**), Spi-GO (3:7) (**B**), Met-GO (3:7) (**C**) and Bet-GO (1:9) (**D**).

Thermal stability analysis was carried out by comparing the Thermogravimetric analysis (TGA) curves of GO and pesticide-GO nanocomposites (Figure 4). GO showed a weight loss of 27.72% at 200 °C, while Chl-GO, Spi -GO, Met-GO and Bet-GO nanocomposites were stable with a weight loss of 6.03%, 7.22%, 2.34% and 3.37%, respectively, showing a better thermal stability after GO loading with pesticides.

By comparing the curves of Chl, Spi, Met and Bet, the weight loss at 200 °C was 2.37%, 43.70%, 2.10%, 0.00%, respectively, indicating that the thermal stability of GO-insecticide nanocomposites was higher than that of GO, but lower than that of Chl, Spi, Met and Bet. This phenomenon was related to the weight loss of oxygen-containing functional groups in GO, according to the structure Chl, Spi, Met and Bet hardly lost functional groups. Chl-GO, Spi-GO, Met-GO and Bet-GO nanocomposites occupied part of the oxygen-containing functional groups due to the H-bond interaction between the pesticides and GO.

### 3.4. FT-IR Spectra of GO-Pesticide Nanocomposites

**Figure 5:**
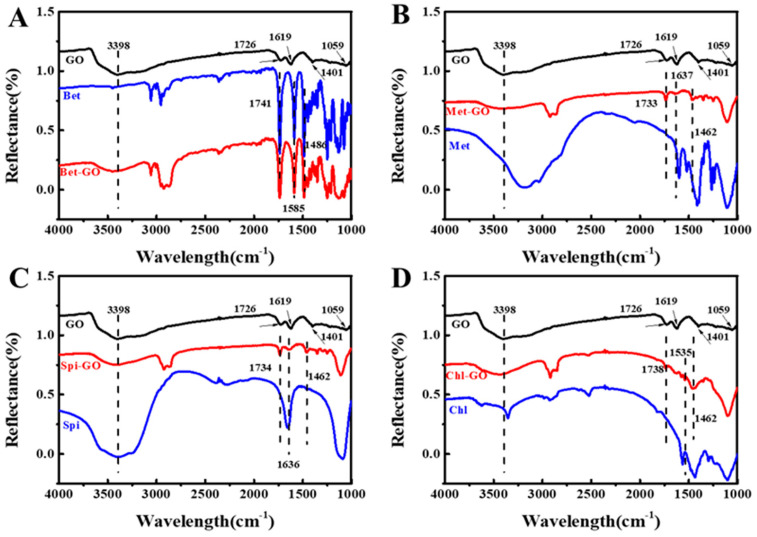
FT-IR spectra of Bet-GO at (1:9) (**A**), Met-GO at 3:7 (**B**), Chl-GO at 5:5 (**C**), Spi-GO at 3:7 (**D**) characterization of GO, pesticide and pesticide-GO.

The functional groups of GO and pesticide GO nanocomposites were investigated by FTIR spectra, as shown in Figure 5. GO showed characteristic peaks at 3398 cm^−1^, 1726 cm^−1^, 1619 cm^−1^, 1401 cm^−1^ and 1059 cm^−1^. Among them, 3398 cm^−1^ was absorption to the stretching of O-H, and the two typical absorption peaks at 1726 cm^−1^ and 1619 cm^−1^ corresponded to carbonyl C=O and the vibration of sp^2^ carbon skeleton network [32,33], respectively. While the bands at 1401 cm^−1^ and 1059 cm^−1^ may be related to the C-O vibration of various oxygen-containing groups, such as epoxy and carboxyl groups [34]. These groups were conducive to the miscibility and dispersion of GO in the polymer matrix through hydrogen bond or electrostatic interaction.

In the Bet spectrum (Figure 5A), the stretching vibration peaks of benzene skeleton were recorded at 1588 cm^−1^ and 1486 cm^−1^. Similarly, Chl, Met and Spi had stretching vibration of benzene skeleton at 1535 cm^−1^ and 1462 cm^−1^, 1637 cm^−1^ and 1462 cm^−1^, 1636 cm^−1^ and 1462 cm^−1^, respectively. In addition, the four pesticides had stretching vibration of carbonyl at 1741 cm^−1^, 1738 cm^−1^, 1733 cm^−1^ and 1734 cm^−1^, respectively. It can be seen that the FTIR spectra of Bet-GO, Chl-GO, Met-GO and Spi-GO nanocomposites had the characteristic peaks of GO and Bet, Chl, Met and Spi at the same time (Figure 5A–D). The FTIR spectroscopy results were similar to previous studies in literature on GO, CS and GO-CS [35,36]. GO-CS composites arose because of the reactions of epoxy functional groups and amino (NH_2_) groups outlined on the GO surface towards the CS exterior [37]. According to FTIR spectroscopy, it was confirmed that EDTA was successfully loaded on the GO surface [38]. These results further indicated the adsorption of four pesticides on GO through physical interaction.

### 3.5. HPLC Analysis of GO-Pesticide Nanocomposites

The loading capacities of pesticides on GO were determined using the HPLC method. Loading content of pesticides on GO showed that the LC of Spi, Met, Bet and Chl on GO were 53.4%, 67.9%, 68.5% and 69.2%, respectively (Figure S1).

### 3.6. Larval Toxicity Bioassay with Pesticides

The indoor toxicity test results of different chemical pesticides on the third instar larvae of *S. frugiperda* were shown in Table 2. Spi, Chl and Bet were indicated as having good effects on *S. frugiperda* larvae. Among five different types of pesticides, Spi showed the best toxicity, and following were Spi, Chl, Bet and Met.

### 3.7. Optimal Ratio of GO and Pesticides

**Figure 6:**
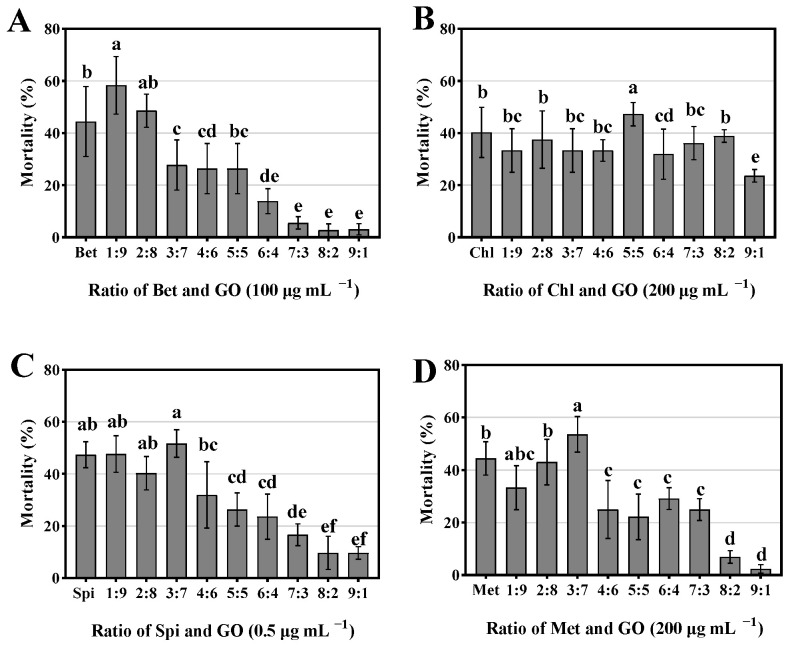
Mortality of *S. frugiperda* upon treatments with different pesticides or with different mass ratios in combination with GO. Mortality rates of Spi and GO (**A**), Chl and GO (**B**), Bet and GO (**C**), Met and GO (**D**) under different mass ratios. Data are mean ± standard error (SE). Error bars represent the SE (N = 3). Different lowercase letters indicate significant differences among treatments (Fisher’s protected LSD test, *p* < 0.05).

In order to screen the best combination ratio of GO and each insecticide, the mortality of *S. frugiperda* was tested when treated by the nanocomposites formed by mixing GO and insecticide with different mass ratios. As shown in Figure 6A, the mortality of *S. frugiperda* under treatments of individual Bet was 44.45%. Mortality reached 58.33% under treatment with Bet-GO combined at a mass ratio of 1:9 as the optimal combination ratio of Bet and GO. Chl-GO combined at the ratio of 5:5 showed higher efficacy than other combinations (Figure 6B), and Spi-GO combined at the ratios of 1:9 and 3:7 resulted in the highest mortality (Figure 6C). Considering the cost and feasibility in practice, we chose Spi-GO, Met-GO, Bet-GO and Chl-GO combined at 3:7, 3:7, 1:9 and 5:5, respectively, as the optimal combinations in bioassay analysis and synergy assay.

### 3.8. Bioassay of GO-Pesticide Nanocomposites

**Figure 7:**
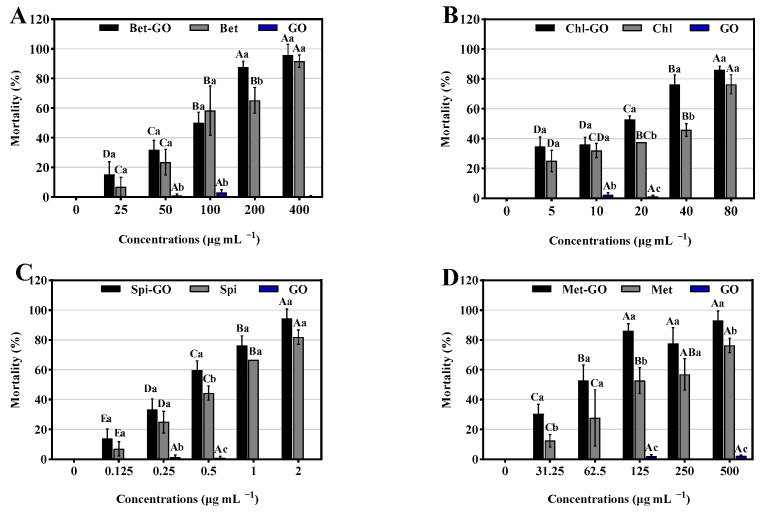
The synergistic effect of GO on the control of the third instar larvae of *S. frugiperda* by Bet (**A**), Chl (**B**), Spi (**C**) and Met (**D**). Different lower case letters indicate significant differences between treatments within the same concentration; different capital letters indicate significant generational differences at the same treatment level (Fisher’s protected LSD test, *p* < 0.05). Data are mean ± standard error (SE). Error bars represent the SE (N = 3).

The synergistic effects of GO on Bet, Chl, Spi and Met on the third instar larvae of *S. frugiperda* were shown in Figure 7. Mortalities of *S. frugiperda* treated with different concentrations of Bet after 48 h were 6.94%, 23.61%, 58.33%, 65.28% and 91.67% (Figure 7A). In the combination of GO and Bet, the concentration of Bet-GO mixed agent was 200 μg/mL, which had the most significant control effect on *S. frugiperda*. Furthermore, the synergistic effect of GO on the contact toxicity of Chl was investigated, and the mortality values of *S. frugiperda* was 25.00% to 75.39% at different concentrations of Chl. After treated with Chl-GO mixture, the mortality values of *S. frugiperda* was 35.00% to 86.11% which increased more than 10% to Chl with significant synergistic effects in 40 μg/mL (Figure 7B). Under Spi treatment, the mortality was 6.94% to 81.94%. Under the same concentration of Spi-GO, the mortality rate increased to the range of 13.89% to 94.44% (Figure 7C). The mortality values of *S. frugiperda* ranged from 12.50% to 72.39% under Met treatment. It ranged from 30.56% to 96.06% after Met GO treatment, that is 18.06% higher than that of single Met. Significant synergistic effects were found in 125 μg/mL (Figure 7D). In our experiment, 2 mL DMSO and 0.1% tween-80 was used as adjuvant to improve the dispersibility of pesticide. No mortality was found among the third instar larvae which was treated with distilled water containing 2% DMSO and 0.1% Tween-80 after 48 h, while the larvae treated with GO had at less than 5% mortality. It indicated that GO had no insecticidal effect on *S. frugiperda.* This result was similar to that of Wang et al. [23], indicating that GO had good biocompatibility when used as a synergist.

Bioassays were carried out to determine LC_50_ values of pesticides and GO-pesticide nanocomposites against *S. frugiperda* at 48 h. LC_50_ of Chl was 14.12 μg/mL, whereas the LC_50_ of GO-Chl nanocomposites was 11.00 μg/mL. It indicated that GO could increase the insecticidal activity of Chl by 1.56 times. Similarly, GO can increase the insecticidal activity of Bet, Met and Spi by 1.54, 2.53 and 1.74 times, respectively. There was a statistically significant difference between all complexes and single dose (*p* < 0.05), also indicating that the mixture of GO and pesticides could increase the efficacy while reducing the use of pesticides (Table 3).

## 4. Discussion

This study aimed to determine the synergistic effect of GO-insecticide on the third instar larvae of *S. frugiperda*, which mainly relied on synthetic insecticides and biological insecticides. However, the use of synthetic pesticides damaged the environment and led to resistance to pesticides [39,40]. It presented significant challenges in controlling this pest insect, so there was a need to come up with a subtle but more effective method in controlling *S. frugiperda*. It did not pose a danger to the environment and at the same time it did not develop resistance to the insect. GO has been proven to be the most promising pesticide carrier. The application of GO will not only cause no harm to the environment, but will also minimize the use of pesticides for high efficacy.

The synergistic mechanism of GO and pesticides may relate to the fact that GO can cause mechanical damage to the insect body skeleton, causing the insect to lose water rapidly. The damaged body wall provides a new channel for the penetration of pesticides into insect, and the GO adsorbed by the pesticide can adhere to the insect body wall and improve the pesticide utilization [23]. Determination of loading capacity of graphene oxide to pesticides is related to the structure of pesticides. The four pesticides selected in this study had different structures, so the loading capacity of GO was different. The π-π conjugation between the aromatic ring in the pesticide structure and GO promoted the adsorption of pesticides by GO. Spi does not contain aromatic ring. Met, Bet and Chl contain two aromatic rings, so the drug loading of GO on Met, Bet, Chl was significantly higher than Spi [41]. Since Met, Bet and Chl contained two benzene rings, similar to the π-π conjugation of GO, there is no difference in the adsorption capacity of GO to them.

In addition, our result was similar to Devaj et al. (2021), who tried to combine graphene oxide with malathion and endosulphan to control *Aedes aegypti*. It showed that the control effect on *A. aegypti* could be improved in the early fourth instar and combination of GO-endosulphan was better in effect [42]. To control Asian corn borer, GO combined with pesticides can increase the contact toxicity by 2.10, 1.51 and 1.83 times [23]. GO and the three acaricides (pyridaben, chlorpyrifos and beta-cyfluthrin) against *Tetranychus truncatus and Tetranychus urticae* can significantly increase the contact toxicity by 1.77, 1.56 and 1.55 times, separately, compared with acaricide alone [16].

At the same time, by loading GO with hymexazol modified polydopamine, the prepared nanopesticide can improve the absorption and utilization rate of hymexazol on plants. Through a simulated rain wash, the adhesion of the nanopesticide on plants was found better than that of the hymexazol solution or the hymexazol solution added with surfactant, and it has a better control effect on Cucumber *Fusarium* wilt [43]. The aim of compounding GO with pesticides was to minimize the use of pesticides. Interestingly, more studies have shown that combining bioactive components with nanomaterials can not only increase the toxicity against the target pests, but also reduce volatility for a longer sustained period, thereby improve the efficiency of low-dose pesticides [44,45].

GO modified with copper selenide can preserve 400% (*w*/*w*) of pesticides and remain in the reservoir in the leaf without drift loss, while graphene oxide combined with copper selenide forms a complex with photothermal and photocatalytic, and has a drift resistance property [46]. Polyethylene glycol-coated garlic essential oil nanoparticles were used to control *Tribolium castaneum*, and its control effect reached over 80%, while garlic essential oil alone at the same concentration was only 11% of control efficiency [47]. Similarly, *Spodoptera litura* and *Achaea janata* larvae decreased feeding on castor leaves treated with α-pinene and linalool-encapsulated silica dispersed in acetone, leading to insect starvation and death [48]. All these pesticide combined cases suggested the increased utilization of pesticides.

## 5. Conclusions

In this study, GO was used as a nanocarrier to load four pesticides, and the pesticide-GO (Chl-GO, Bet-GO, Spi-GO, Met-GO) nanocomposite was prepared to show strong synergistic effects on *S. frugiperda* control. The pesticide was adsorbed on the surface of graphene oxide by physical adsorption, indicating the good thermal stability. Compared with single-agent pesticides, pesticide-GO can reduce the pesticide quantity by improving the utilization rate to achieve equivalent control effects. As this research is still at its early exploratory stage, the concentration of pesticide-GO, commercial preparations and the toxicity of pesticide-GO to variant populations still need to be further explored. Therefore, nano pesticides require continuous explorations in large-scale industrialization, commercial production and field application.

## Figures and Tables

**Table 1:** Chl, Bet, Spi and Met alone and combined GO bioassayed against *S. frugiperda*.

Treatment	Concentrations (μg/mL)
CK	2%DMSO, 0.1% Tween 80 and water solution
Chl, GO, Chl-GO	5, 10, 20, 40, 80
Bet, GO, Bet-GO	25, 50, 100, 200, 400
Spi, GO, Spi-GO	0.125, 0.25, 0.5, 1, 2
Met, GO, Met-GO	31.25, 62.5, 125, 250, 500

Mortality = The number of dead insects/the total number of insects tested × 100%.

**Table 2:** Toxicity of 4 pesticides to 3rd instar larvae of *S. frugiperda* at 48 h.

Treatment	Instar	Slope ± SE ^a^	χ^2^	LC50 (95%FL) ^b^	LC90 (95%FL)	df
Spinetoram	3rd instar	1.81 ± 0.20	3.47	0.49	3.96	12
				(0.41–0.60)	(2.61–7.44)	
Methoxyfenozide	3rd instar	1.40 ± 0.15	7.22	159.88	1746.32	15
				(126.46–208.20)	(1023.91–3928)	
Chlorantraniliprole	3rd instar	1.40 ± 0.17	4.96	14.12	199.5	13
				(11.17–18.66)	(113.02–458.75)	
Beta cypermethrin	3rd instar	2.20 ± 0.21	8.25	103.69	578.64	13
				(84.04–128.07)	(395.41–1042.74)	

^a^ is the slope of the linear equation ± standard error. ^b^ LC_50_ values and 95% confidence limits (CL). n = 360 (The number of larvae treated) Biological repeat 3 times.

**Table 3:** Synergistic effect of GO on four pesticides against the third instar larvae of *S. frugiperda* at 48 h.

Treatment	Slope ± SE ^a^	χ^2 b^	df	N ^c^	LC_50_ (95% FL) ^d^	SR ^e^
Chl	1.40 ± 0.17	4.96	13	360	14.12 (11.17–18.66)	-
Chl-GO	1.32 ± 0.17	7.16	13	360	9.00 (8.32–13.98)	1.56
Bet	2.20 ± 0.20	8.25	13	360	104.70 (84.04–128.07)	-
Bet-GO	2.38 ± 0.22	6.63	13	360	68.00 (64.11–94.08)	1.54
Met	1.40 ± 0.15	7.22	15	360	159.88 (126.46–208.20) 63.14	-
Met-GO	1.59 ± 0.21	9.30	13	360	(49.84–77.56)	2.53
Spi	3.80 ± 0.48	3.47	12	360	0.49 (0.41–0.60)	-
Spi-GO	2.12 ± 0.20	8.61	13	360	0.28 (0.24–0.33)	1.74

^a^ is the slope of the linear equation ± standard error. ^b^ Goodness-of-fit test. ^c^ The number of larvae treated. ^d^ LC_50_ values and 95% confidence limits (CL). ^e^ SR is synergism ratio at LC_50_ values. Biological repeat 3 times.

## Data Availability

Not applicable.

## Supplementary Materials

The following supporting information can be downloaded at: https://www.mdpi.com/article/10.3390/nano12223985/s1, Figure S1: HPLC standard curves of four pesticides.
