# Supplementary material for: Synergistic Effects of Graphene Oxide and Pesticides on Fall Armyworm, Spodoptera frugiperda
Source: Nanomaterials (Basel). 2022 Nov 12;12(22):3985. doi: 10.3390/nano12223985 (PMC9692536; doi:10.3390/nano12223985)
Supplement: Supplementary file 1 [file nanomaterials-12-03985-s001.zip › nanomaterials-1979778-supplementary.pdf]

Supporting Information

Synergistic effects of graphene oxide and pesticides on fall armyworm, *Spodoptera frugiperda*

Xue Li[1,2], Qinying Wang[2], Xiuping Wang[3], Zhenying Wang[1*]

[1] State Key Laboratory for Biology of Plant Diseases and Insect Pests, Institute of Plant Protection, Chinese Academy of Agricultural Sciences, Beijing 100193, China

[2] Plant Protection College, Hebei Agricultural University, Baoding 071000, China

[3] Analysis and Testing Center, Hebei Normal University of Science and Technology, Qinhuangdao 066000, China

[*] Correspondence: zywang@ippcaas.cn;Tel. + 86 10 62815945,

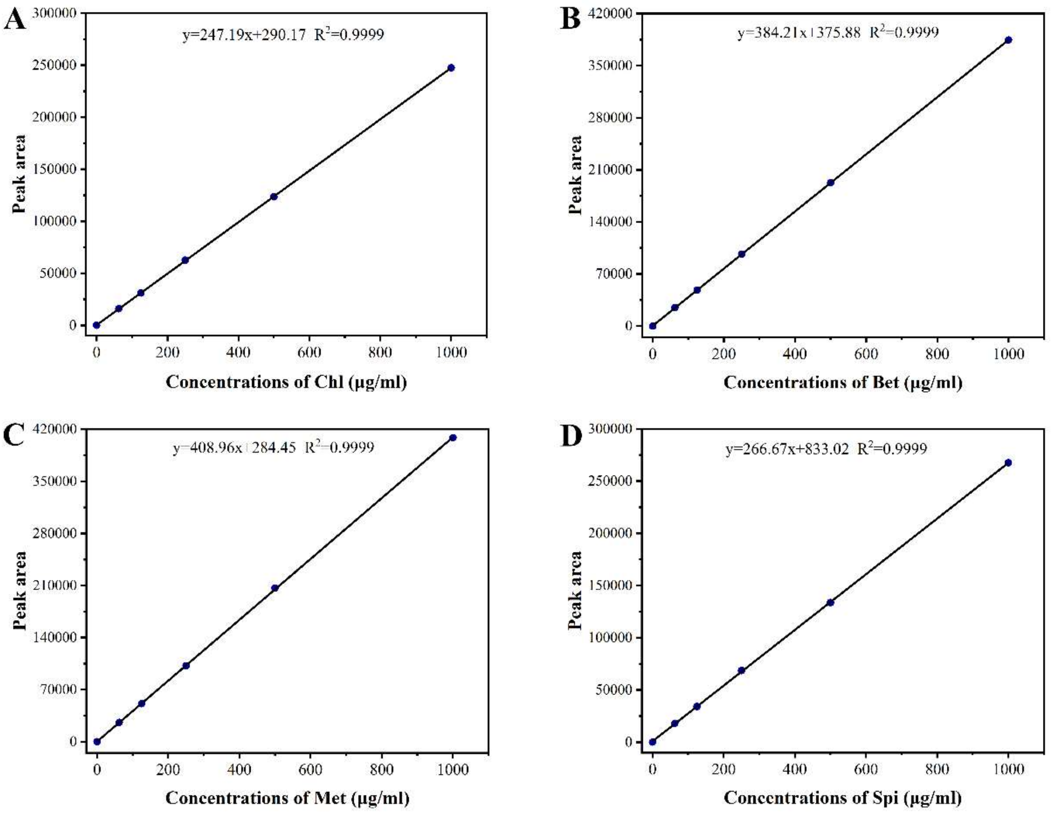

Figure S1. HPLC standard curves of four pesticides.
